# Intercepting a sound without vision

**DOI:** 10.1371/journal.pone.0177407

**Published:** 2017-05-08

**Authors:** Tiziana Vercillo, Alessia Tonelli, Monica Gori

**Affiliations:** 1 Department of Psychology, University of Nevada, Reno, Nevada, United States of America; 2 U-VIP, Unit for Visually Impaired People, Fondazione Istituto Italiano di Tecnologia, Genoa, Italy; Emory University, UNITED STATES

## Abstract

Visual information is extremely important to generate internal spatial representations. In the auditory modality, the absence of visual cues during early infancy does not preclude the development of some spatial strategies. However, specific spatial abilities might result impaired. In the current study, we investigated the effect of early visual deprivation on the ability to localize static and moving auditory stimuli by comparing sighted and early blind individuals’ performance in different spatial tasks. We also examined perceptual stability in the two groups of participants by matching localization accuracy in a static and a dynamic head condition that involved rotational head movements. Sighted participants accurately localized static and moving sounds. Their localization ability remained unchanged after rotational movements of the head. Conversely, blind participants showed a leftward bias during the localization of static sounds and a little bias for moving sounds. Moreover, head movements induced a significant bias in the direction of head motion during the localization of moving sounds. These results suggest that internal spatial representations might be body-centered in blind individuals and that in sighted people the availability of visual cues during early infancy may affect sensory-motor interactions.

## 1. Introduction

Sound localization is an important aspect of our hearing as it facilitates interactions with environmental stimuli. Within real environments, sound sources are not always static but can be dynamic, and accounting for these spatial changes over time becomes crucial for an accurate localization. Furthermore, humans are meant to move, so the big challenge for the brain is to extrapolate spatial positions of moving objects and preserve these representations despite self-movements.

Identifying sound source locations becomes particularly relevant when external stimuli are not accessible to the visual system; for example, when stimuli are located out of the visual field (as in the case of a car approaching from behind) or, more importantly, in the case of visual impairment. A number of studies reported enhanced hearing abilities in blind individuals after early visual deprivation, suggesting that the brain might take advantage of the auditory modality to compensate for the lack of vision [1–4]. Some forms of sensory compensation seem to be driven by a cortical reorganization of the visual cortex, which preserves its computational processing but develops sensitivity to non-visual stimuli [5–11]. Other recent studies reported task-specific spatial auditory impairment in blind individuals [12–15], supporting the idea that the visual system might be important for the spatial calibration of the auditory space [16], [17]. Most of these studies used static auditory stimuli paired with a static body position of the participant, neglecting more natural conditions that consider body movements and dynamic auditory stimuli.

The localization of moving auditory stimuli may be poorly accurate, as object in motion can be displaced toward the direction of motion [18–20]. This spatial displacement depends on a number of variables [19] such as target velocity [20], predictability and position of the target [21], type of visual motion and response mode [22]. Although the idea of a perceptual spatial distortion for moving objects might seems detrimental, such displacement sometimes plays an important role for the organization of goal-directed movement. For instance, to catch a target in motion the perceptual system must compensate for the target’s movement occurring during visual processing and during the phase of motor preparation. Under this perspective, a spatial displacement may reflect the ability of the brain to predict the future position of the target and facilitate rapid and accurate motor responses, binding the gap between perception and action [19], [23]. This hypothesis is consistent with “forward models” suggesting an internal mechanism triggered by the efferent copy of the motor command that predicts sensory consequences to improve sensory-motor interaction [24–26].

The localization of visual stimuli or sound sources apparently doesn’t change under dynamic conditions [27], [28]. In fact, people make no directional errors when have to report the final location of a moving auditory target after rotational movement of the head. Perceptual stability occurs through a spatial remapping of the stimulus from an egocentric (eye, head and body centered coordinates) to an allocentric, i.e. external, frame of reference that guarantees a stable representation of objects in world coordinates. In the acoustic domain, external stimuli are represented in both egocentric and allocentric frames of reference [29]. Indeed, the egocentric spatial representation of sound sources that is originally deduced from the processing of binaural cues such as interaural time difference (ITD) and interaural level difference (ILD) afterward is remapped in an allocentric frame of references, to ensure an accurate multisensory perception and sensory-motor interaction.

To overcome the limits of using static auditory stimuli and static body positions of participants, we tested a group of sighted and early blind individuals in different auditory tasks to investigate auditory localization of static and moving sound sources with and without head movements. This study explores: i) audio processing in dynamic contexts; ii) the role of body movement on sound perception and iii) the association between auditory processing and body movement (in terms of reference systems and interaction abilities). As mentioned above, moving stimuli are usually displaced toward the direction of motion and this perceptual illusion might be important for sensory-motor interaction. Head movements affect binaural cues adopted by the auditory system for localization and these changes may result in a spatial displacement. Nevertheless, if self-movements trigger the spatial remapping of the sound in allocentric frames of reference, the perceived location of the sound should not change. Our hypothesis is that visual deprivation might affect the localization of static and dynamic auditory stimuli and the remapping into allocentric coordinates that usually occurs during head movements.

## 2. Methods

Eight healthy volunteers (4 males, 4 females, mean age: 36 ± 6 years of age) and eight early blind individuals (3 males, 5 females, mean age: 40.12 ± 6 years of age) participated in the experiment. S1 Table (Supplemental material) shows additional details about the age, pathology and residual vision of the blind participants. All participants were right handed and had normal hearing. Sighted participants were blindfolded during the experiment. The study was conducted according to the principles defined in the declaration of Helsinki. All testing procedures were approved by the ASL3 of Genoa (Italy). Participants provided signed informed consent after the experimental procedures were explained.

**Fig 1:**
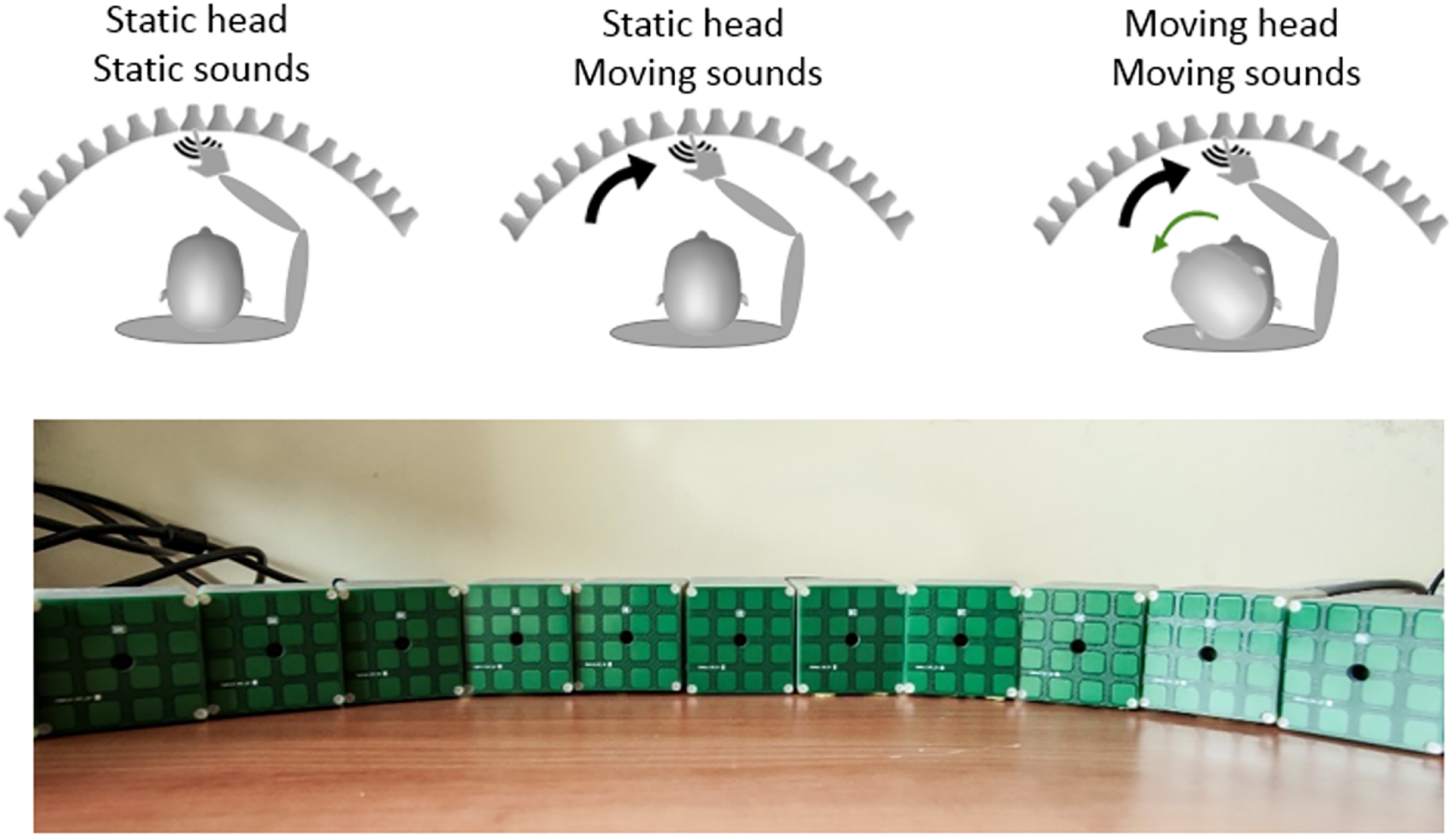
Upper panels shows the procedures for the three different localization tasks. The lower panel shows a picture of the experimental setup.

The experimental setup was composed by 18 speakers placed at 5 cm distance one from another and arranged in an arc with 57 cm radius (see Fig 1). Each speaker was covered with 4X4 array of tactile sensors, used to record participants’ responses. Participants sat in the middle of the array. Auditory stimuli were static or moving sounds (white noise burst), presented at 70 dB of sound pressure level. We recorded motor responses by using tactile sensors directly attached to the speaker surface and head movements by using the Vicon motion tracking system (Vicon Motion Systems, Ltd., UK). This is an infrared marker-tracking system that acquires live movements in 3D space with high temporal and spatial precision.

Participants performed three different auditory tasks in a random order: 1) a simple pointing task with static sound sources to control for any bias in sound localization, 2) a localization task with moving sounds, 3) a localization task with moving sounds while participants performed a head movement (see Fig 1 for methods and procedures). During the localization task with static auditory stimuli (task 1), a 300 ms sound was delivered by one of the 18 speakers from the setup. Participants had to identify and touch the speaker that produced the sound. We run a single block where every sound location was repeated 10 times, in a random order, for a total of 180 trials.

In the localization task with moving sounds sources (task 2), participants kept their head straight while listening to a sound moving from left to right or from right to left. While the space displacement of the moving sound was fixed at 15 cm across the experiment, the duration of the sound, and consequently its velocity, was manipulated across three values: 200, 300 and 500 ms where the duration is inversely proportional to the velocity. After the presentation of the moving sound, participants had to locate its endpoint by touching the last speaker that played the sound. We manipulated the direction of sound motion generating a “rightward” and a “leftward” condition. For each direction of the moving sound, participants performed three experimental blocks: one for each stimulus duration. The endpoint of the moving sound ranged between -7.5, 2.5, 12.5, 22.5 and 32.5 cm (given the fixed spatial displacement of the moving sound its start-point ranged between -27.5, -17.5, -7.5, 5 and 12.5 cm) in the rightward condition, where negative values represent the left side and positive values the right side of the array, and -32.5, -22.5, -12.5, -2.5, 7.5 for the leftward condition. Each endpoint location was repeated 10 times in a constant stimuli algorithm, in a random order, for a total of 100 trials.

In the localization task with moving sounds and head movements (task 3), we used the same auditory stimuli of task 2, but this time participants had to perform a head rotation during sound presentation. Specifically, we asked participants to rotate their head in the opposite direction of sound motion (for example in the rightward condition, while the sound was moving to the right the head was rotating to the left side) and to start the movement after a go signal simultaneous to the start of the sound. At the end of the auditory stimulation, participants had to maintain the head rotated and localize the endpoint of the moving sound by touching the speaker with their right hand. Also in this task, participants performed one experimental blocks for each stimulus duration (three blocks).

Before the experiment, we trained participants to perform precise head movements. We analyzed head movements with the Vicon motion capture system. For an accurate analysis, we used seven markers: three of them were placed on participants’ shoulders to form a horizontal line, two markers were placed above the ears, one on the forehead and one above the inion. These last two markers generated a vertical line on the antero-posterior axes of the brain. We measured the intersection between the horizontal and the vertical line and calculated the amplitude of the angle produced by the rotation of the head and the speed of the head movement.

## 3. Results

**Fig 2:**
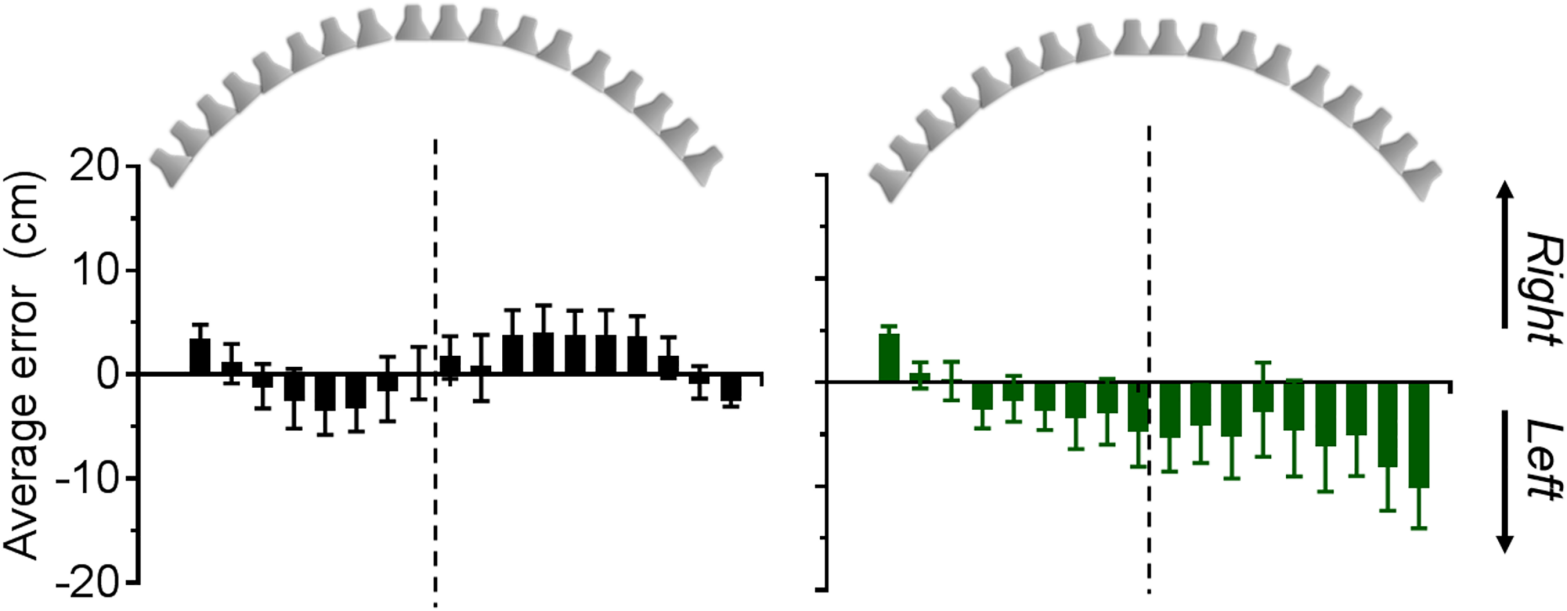
Average errors for each speaker location (locations are represented up in the figure) for the sighted (black bars, left panel) and blind group (green bars, right panel) measured in the localization task with static sounds.

Results from the pointing task with static sound sources are showed in Fig 2. Average errors, calculated for each subject as the difference between the reproduced and the real location of the sound and then averaged across participants for each group, are plotted for each speaker location. Positive values represent a mislocalization to the right, while negative value a displacement to the left. The dashed line represents the central position of the speaker array, so that bars on the right side of the line denote speakers on the right side of the array and bars on the left side of the line denote speakers on the left side of the array. Sighted participants had a small tendency to expand the auditory space, displacing sounds location on the right side of the array to the right (positive errors) and location on the left side of the array to the left (negative errors). However, errors were not statistically different from zero. On the other side, the group of early blind participants showed a significant mislocalization to the left (one sample, two-tailed t-test: t_(17)_ = -4.53, p<0.001). A repeated measure ANOVA revealed a significant effect of the speaker location (F_(17)_ = 4.43, p<0.0001, η^2^ = 0.38). Specifically the error in localizing the last speaker on the right was significantly higher than all the others (all p<0.01).

**Fig 3:**
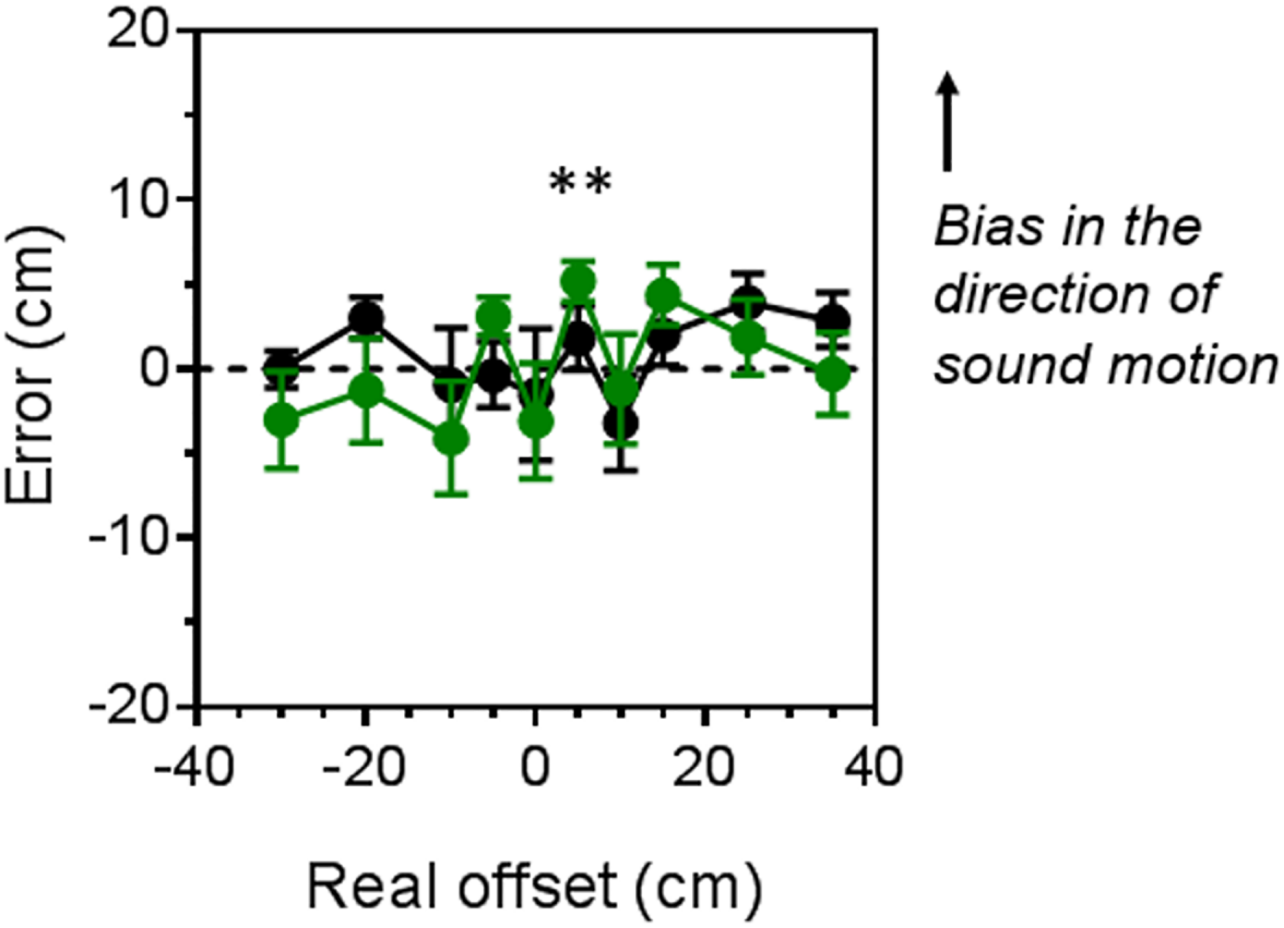
Average errors (from the bias observed in task 1) measured in the localization task with moving sounds and static head for the sighted (black lines and symbols) and blind (green lines and symbols) groups of participants. Data are averaged across the three speed conditions (200, 300 and 500 ms).

Localization errors in task 2 were analysed with a repeated measure ANOVA (within factors: motion direction, speed and endpoint location; between factor: group). For this task, errors were calculated as the difference between reproduced endpoint locations measured in task 2 and reproduced locations measured in task 1. Moreover, we normalized errors to correct for the direction of motion so that positive values of the error represent a bias in the direction of sound motion. Since endpoint locations were specular for the two motion direction conditions, we considered 5 endpoint locations from 1 (most central) to 5 (most peripheral). We found a significant interaction between endpoint location and group (F_(1,2,4,1)_ = 4.11, p = 0.006, η^2^ = 0.25), but no effect of speed and motion direction. Fig 3 shows the results for task 2 for sighted (black symbols and line) and blind (green symbols and line) participants. As we did not find any significant effect of speed, we averaged individual data across the three speed conditions. Group mean errors are plotted as a function of all the endpoint locations of the sound. Positive value of the error represent a displacement toward the direction of sound motion. On average error were significantly different from zero (showing a bias in the direction of sound motion) only for blind participants when the sound ended 5 cm to the right (one sample, 2-tailed t-tests with Bonferroni correction for multiple comparison, t_(7)_ = 4.73, p = 0.002). Sighted controls’ error when the sound ended 25 cm to the right was only marginally significant (one sample, 2-tailed t-tests with Bonferroni correction for multiple comparison, t_(7)_ = 2.44, p = 0.04).

**Fig 4:**
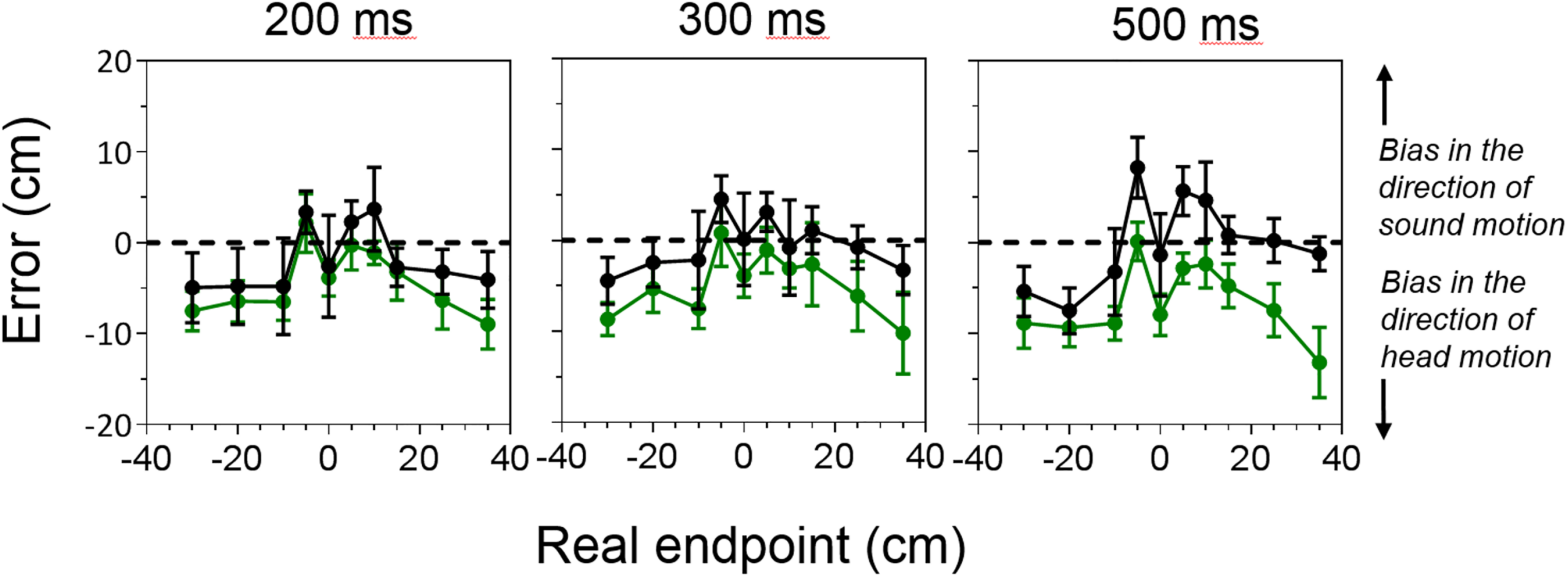
Average errors (from the bias observed in task 1) measured in the localization task with moving sounds and moving head for the sighted (black lines and symbols) and blind (green lines and symbols) groups of participants, for the three speed conditions (200, 300 and 500 ms).

The head rotation, performed in task 3, differently affected the performance of the two group of participants, with blind individuals showing a bias in the direction of head motion. Results for the localization tasks with moving sounds and moving head are reported in Fig 4. Group mean errors are calculated as the difference between reproduced endpoint locations measured in task 3 and errors measured in task 1. Errors were normalized to correct for the direction of motion so that positive values of the error represent a bias in the direction of sound motion and negative values of the error show a bias in the direction of head motion. A repeated measure ANOVA (within factors: motion direction, speed and endpoint location; between factor: group) showed a significant interaction between motion direction, endpoint location and group (F_(1,2,4,1)_ = 4.12, p = 0.008, η^2^ = 0.31); motion direction and endpoint location (F_(1,2,4,1)_ = 8.9, p<0.001, η^2^ = 0.49); speed and endpoint location (F_(1,2,4,1)_ = 3.28, p = 0.003, η^2^ = 0.26); endpoint and group (F_(1,2,4,1)_ = 3.42, p = 0.01, η^2^ = 0.27); and a significant effect of endpoint location (F_(1,2,4,1)_ = 13.72, p<0.001, η^2^ = 0.6). Pairwaise comparison (corrected for multiple comparison) revealed that the mislocalization of peripheral speakers was larger than the mislocalization of the most central speakers (p<0.002).

For the rightward condition, differences in localization’s error between the two groups of participants were significant only for the 500 ms condition (repeated measure ANOVA, group for the 500 ms condition; within factors: endpoint location; between factor: group, F_(4,1)_ = 9.28, p = 0.009, η^2^ = 0.39). We also compared localization error measured in this task with localization errors observed in the task with moving sound and static head. A repeated measure ANOVA (within factor: head, speed and endpoint; between factor: group) showed a significant effect of head (F_(1,3,4,1)_ = 8.44, p = 0.01, η^2^ = 0.37) and endpoint (F_(1,3,4,1)_ = 6.57, p<0.001, η^2^ = 0.44), a significant interaction between head and group (F_(1,3,4,1)_ = 4.66, p = 0.04, η^2^ = 0.25) and head and endpoint (F_(1,3,4,1)_ = 15.09, p<0.001, η^2^ = 0.40). Similarly to the rightward condition, for the leftward condition, a repeated measure ANOVA (within factor: head, speed and endpoint; between factor: group) showed a significant interaction between endpoint and group (F_(1,3,4,1)_ = 3.88, p = 0.01, η^2^ = 0.30) and head, endpoint and group (F_(1,3,4,1)_ = 3.49, p = 0.01, η^2^ = 0.28).

## 4. Discussion

Results from this study showed that early blind individuals are inclined to displace static sounds toward the left side of space. Results also showed that rotational head movements affect auditory localization in early blind individuals but not in sighted people. Overall, these findings suggest that early visual deprivation has an effect on auditory localization and that auditory spatial representations in blind individuals may be body-centered.

In the localization task with static sound, blind participants showed a spatial bias to the left. This result might appear in conflict with previous study reporting similar or even higher auditory spatial accuracy in blind as compared to sighted individuals (Lessard et al., 1998; Röder et al., 1999). We believe that the high spatial resolution provided by our setup (the distance between the center of two adjacent speakers was 5 cm) enabled us to highlight even small spatial errors that otherwise might not be detectable. Indeed, in a previous study (Lessard et al., 1998) the distance between speakers was 10 cm, which is the largest error that we reported in this task.

By positioning speakers on a table (not at the ear level) we might have added reverberation and affected sound perception. On the other hand, reverberation should have equally impaired the performance of both group of participants, and not only that one of the blind group. Other studies support our result, showing a leftward bias in blind individuals for a tactile bisection task [30] that might depend on differences in the role of the right and left hemisphere in the control of spatial attention [31].

Stimulus motion induced a small spatial bias in both groups, as previously reported in the visual modality [18], [19] and in the auditory modality [20]. Interestingly, blind and sighted participants showed opposite trend with blind individuals displacing central stimuli and sighted controls only peripheral sounds. This might be explained by an enhanced auditory localization for peripheral stimuli in blind individuals [3].

The more interesting result of the current study is that rotational head movements impaired sound localization in blind individuals, inducing a bias in the direction of head motion, but did not affect the performance of sighted participants as previously reported [28]. This result suggests that in early blind, the spatial remapping of the auditory stimulus in an allocentric frame of reference does not occur and that blind participants might be more susceptible to a motor bias. An egocentric representation of the surrounding space may be extremely functional for blind individuals during navigation as it provides an additional “margin of safety” and can help to avoid obstacles [32]. Previous studies already supported an egocentric spatial representation in blind individuals [33], [34]. This result is also consistent with a recent study from Finocchietti et al. [12] reporting a deficit in blind individuals in encoding sound motion in the lower side of the sagittal plane with a spatial bias toward the head.

We propose that vision plays a crucial role in organizing auditory spatial processing. Developmental studies already showed that before 10–14 years of age, audio-visual and visual-haptic stimuli are not integrated optimally as in adults, but vision dominates over audition and touch for spatial judgments [16], [35], supporting the idea that vision is important for the spatial calibration of the other sensory modalities [17]. In agreement with recent findings [12–14], [36–38], here we showed that the presence of visual cues during the early infancy affects auditory spatial representations.

Vision, over all sensory modalities, is usually defined as a spatial sense [39] because it represents proximal and distal items simultaneously, extracts spatial invariants despite self-motion and provides the most precise information about consequences of self-displacement. The severe reduction of distal information and the poor representation of external landmarks induced by the loss of vision may prompt the use of egocentric frames of reference because allocentric representations become more difficult to process. Indeed, it has been argued that early blind spatial knowledge rely more on body-centered proprioceptive and kinesthetic information (Millar, 1994). In agreement with this hypothesis, we suggest that early visual deprivation shapes internal spatial representations, enhancing the salience of the body as a reference. Our findings suggest that visual information is important not only for a spatial calibration of the other sensory modalities, but also for the correct development of sensory-motor interactions.

## Supporting information

S1 Table Individual age, diagnosis and residual vision for each of the blind participants we have tested. (DOCX)
