# Supplementary material for: Intercepting a sound without vision
Source: PLoS One. 2017 May 8;12(5):e0177407. doi: 10.1371/journal.pone.0177407 (PMC5421809; doi:10.1371/journal.pone.0177407)
Supplement: S1 Table — (DOCX) [file pone.0177407.s001.docx]

# **Table**

We have reported the individual age, diagnosis and residual vision for each of the blind participants we have tested.

| **Impairment** | **Age** | **Gender** | **Diagnosis** | **Residual Vision** |
| --- | --- | --- | --- | --- |
| Blind | 56 | M | Fibroplasia retrolentale | No vision |
| Blind | 49 | M | Retinopathy of Prematurity | No vision |
| Blind | 22 | F | Congenital glaucoma and  retinal detachment | No vision |
| Blind | 54 | F | ICD9 - 362.7  Retinitis pigmentosa (ICDS - 379.3 cheratoplastica, afachia chirurgica) | Ligths and shadows |
| Blind | 25 | F | Retinopathy of Prematurity | No vision |
| Blind | 56 | M | ICD9 - 365.4 Congenital glaucoma | No vision |
| Blind | 27 | F | ICD9 - 362.7  Retinitis pigmentosa | Ligths and shadows |
| Blind | 33 | F | Congenital chataract/Attic atrophy | No vision |
